# PRISMA-DTA for Abstracts: a new addition to the toolbox for test accuracy research

**DOI:** 10.1186/s41512-021-00097-4

**Published:** 2021-04-02

**Authors:** Daniël A. Korevaar, Patrick M. Bossuyt, Matthew D. F. McInnes, Jérémie F. Cohen

**Affiliations:** 1 Department of Respiratory Medicine, Amsterdam University Medical Centres, University of Amsterdam, Meibergdreef 9, 1105 AZ Amsterdam, the Netherlands; 2 Department of Clinical Epidemiology, Biostatistics and Bioinformatics, Amsterdam University Medical Centres, University of Amsterdam, Amsterdam, the Netherlands; 3 Departments of Radiology and Epidemiology, University of Ottawa, Ottawa, Canada; 4 Clinical Epidemiology Program, the Ottawa Hospital Research Institute, Ottawa, Canada; 5 Department of General Pediatrics and Pediatric Infectious Diseases, Necker-Enfants Malades Hospital, Assistance Publique-Hôpitaux de Paris, Paris, France; 6 Inserm UMR 1153, Obstetrical, Perinatal and Pediatric Epidemiology Research Team, Center of Research in Epidemiology and Statistics (CRESS), Université de Paris, Paris, France

## Introduction: reporting guidelines

Complete reporting of biomedical research is essential to ensure that readers can reproduce the study methodology, are informed about quality concerns such as potential sources of bias, and understand to which patients the results are applicable. There are ongoing concerns about the quality of study reports in many fields of biomedical research [1, 2]. Test accuracy research, in which the ability of signs and symptoms, biomarkers, or medical tests to identify a target disease is evaluated, is not exempt from this problem. Numerous evaluations have shown that reports of test accuracy studies and systematic reviews thereof often lack crucial information, mostly about the methods applied and the results found [3]. This leads to research waste and threatens research integrity.

Currently, several hundreds of reporting guidelines are available in the EQUATOR (Enhancing the QUAlity and Transparency Of health Research) network’s library, where researchers can identify which of these is most suitable for their specific study design [4]. The first and most well-known is CONSORT (Consolidated Standards of Reporting Trials) for reports of clinical trials, first published in 1996, and updated several times after this [5]. Since then, reporting guidelines have been developed for many kinds of study designs, including TRIPOD (Transparent Reporting of a Multivariable Prediction Model for Individual Prognosis or Diagnosis) for prediction model studies [6], STARD (Standards for Reporting of Diagnostic Accuracy Studies) for test accuracy studies [7–9], and PRISMA (Preferred Reporting Items for Systematic Reviews and Meta-analyses) for systematic reviews [10]. These reporting guidelines consist of a list of essential items (sometimes referred to as a “checklist”) that should be reported to ensure optimal informativeness and transparency. This will allow for easy identification of the study in online libraries and databases and for adequate assessment of study methodology, applicability, and results. Many reporting guidelines are accompanied by an “Explanation and Elaboration” document, which provides specific and detailed guidance on how to report each essential item on the list, along with examples of good reporting practices.

The recent publication of PRISMA-DTA for Abstracts and its accompanying Explanation and Elaboration document provides a new addition to the toolbox for reporting test accuracy research [11–13]. We here set out which steps led to the development of PRISMA-DTA for Abstracts and which tools are currently available to improve and assess the reporting and the methodological quality of test accuracy research.

## Reporting guidelines for abstracts

Earlier versions of reporting guidelines primarily provided guidance for full-text articles, but there has also been growing attention for reporting journal and conference abstracts over the years. This started with CONSORT for Abstracts, which was published in 2008 as an extension of CONSORT and provided guidance for reporting abstracts of clinical trials [14]. Since then, extensions of other reporting guidelines specifically focusing on the reporting of abstracts have been developed. Currently reporting guidelines for abstracts are available for at least five types of study designs: clinical trials, observational studies, systematic reviews, test accuracy studies, overviews of systematic reviews, and multivariable prediction models [15, 16]. More are likely to follow.

The abstract has become a fundamental part of a study report, which may have a considerable impact on the interpretation of a study for the average reader. Many users of the biomedical literature only read the abstract, either due to time constraints or because they do not have access to the full text. In addition, systematic reviewers and guideline developers rely on accurate information in the abstract because they often need to screen large amounts of them for potential eligibility. Also, if a study is presented at a scientific conference, the abstract is often the only bit of information available about the study, and many studies reported as conference abstracts are never published in full [17].

It has been shown numerous times that reporting in abstracts, also in test accuracy research, is frequently incomplete, which could lead to misinterpretation and overinterpretation of the study findings [18–20]. This may be the case if crucial design elements resulting in potential sources of bias or generalizability concerns are not evident, or if the authors “spin” their findings, which has shown to be more frequent in abstracts than in full texts [21–23].

## STARD 2015 and STARD for Abstracts

Test accuracy studies evaluate the performance of medical tests by comparing their results with a reference standard, where results are expressed in estimates of diagnostic accuracy such as sensitivity and specificity. In 2003, the STARD reporting guideline was published for these studies, and an updated version was launched in 2015 [7–9]. STARD 2015 contains a list of 30 essential items. As for most reporting guidelines, some of these items are “general,” applying to any biomedical study involving patients. However, test accuracy studies have a number of design features and outcomes that are very typical of this type of research. In addition, research has shown that these studies are sensitive to several sources of bias and variation [24]. Items on STARD 2015 that are specific to test accuracy studies are, for example, the instruction to report the intended use and clinical role of the index test (item 3), which reference standard was used and how it was applied (item 10b), the definition of test positivity cut-offs or result categories (item 12), whether test readers were masked (item 13), how missing test data were handled (item 16), and estimates of diagnostic accuracy with confidence intervals (item 24). Evaluations have shown that completeness of reporting improved over the years after the dissemination of STARD [25]. In response to empirical evidence of incomplete reporting of abstracts of test accuracy studies, STARD for Abstracts was additionally published in 2017, providing specific guidance for writing journal and conference abstracts [18, 19, 26].

## PRISMA-DTA and PRISMA-DTA for Abstracts

The PRISMA guideline was first published in 2009 as a guiding tool for authors writing reports of systematic reviews [10]. In 2013, a subsequent extension for abstracts of systematic reviews was published, PRISMA for Abstracts [27]. Although PRISMA can be used as a basis for reporting systematic reviews of any type of research, it mainly focuses on reviews of randomized trials of interventions. With the number of systematic reviews of test accuracy studies growing rapidly over the past years, an extension explicitly focusing on this study design was deemed useful. Like for primary test accuracy research, systematic reviews of test accuracy studies have typical design and results characteristics that are, to some extent, unique to this type of research [28]. This resulted in the PRISMA-DTA reporting guideline, published in 2018 [11, 12].

PRISMA-DTA also provides guidance for reporting abstracts (Table 1). A baseline assessment of adherence to PRISMA-DTA for Abstracts in 100 systematic reviews of test accuracy studies showed that, on average, only 5.5 of 11 guideline items had been reported. Crucial items such as study characteristics used as criteria for eligibility (item 3, reported by 57%), literature search dates (item 4, 42%), characteristics of included studies including the reference standard (item 6, 13%), methods of assessing the risk of bias (item 5, 38%), and study registration number (item 12, 5%) were often not reported in the abstract [29].

**Table 1:** PRISMA-DTA for Abstracts checklist

Section and topic	Item no.	Description
**Title and purpose**		
Title	1	Identify the report as a systematic review (+/− meta-analysis) of diagnostic test accuracy studies.
Objectives	2	Indicate the research question, including components such as participants, index test, and target conditions.
**Methods**		
Eligibility criteria	3	Include study characteristics used as criteria for eligibility.
Information sources	4	List the key databases searched and the search dates.
Risk of bias and applicability	5	Indicate the methods of assessing risk of bias and applicability.
Synthesis of results	A1	Indicate the methods for the data synthesis.
**Results**		
Included studies	6	Indicate the number and type of included studies and the participants and relevant characteristics of the studies (including the reference standard).
Synthesis of results	7	Include the results for the analysis of diagnostic accuracy, preferably indicating the number of studies and participants. Describe test accuracy including variability; if meta-analysis was done, include summary results and confidence intervals.
**Discussion**		
Strengths and limitations	9	Provide a brief summary of the strengths and limitations of the evidence.
Interpretation	10	Provide a general interpretation of the results and the important implications.
**Other**		
Funding	11	Indicate the primary source of funding for the review.
Registration	12	Provide the registration number and the registry name.

The PRISMA-DTA for Abstracts list is also available in the EQUATOR network’s library (https://www.equator-network.org/)

The original length of the PRISMA for Abstracts guidelines was maintained in PRISMA-DTA for Abstracts: it also consists of 12 items. Some items apply to any type of systematic review and were unchanged, such as the key databases searched and the search dates (item 4), the number and type of included studies (item 6), and the primary source of funding (item 11). Eventually, one item (item 8, calling for the description of effect size) was removed as it does not apply to test accuracy studies, one item (item A1, calling for reporting of statistical methods used for data synthesis) was added, and updated phrasing was used in six additional items, reflecting language and methods more typically used in test accuracy research. The PRISMA-DTA group has now published an extensive Explanation and Elaboration document, with detailed guidance along with examples on how to report each item in an abstract [13].

## Other initiatives to improve test accuracy research

With the publication of PRISMA-DTA for Abstracts, the “toolbox” that can be used in the field of test accuracy research is expanding further. The abovementioned reporting guidelines can be used for primary test accuracy studies and systematic reviews thereof (Table 2), which may be evaluations of diagnostic tests, but is also relevant for medical tests used for screening, staging, prognosis, and monitoring. In addition to these reporting guidelines, multiple other tools have been developed over the past years to improve the quality of this type of research.

**Table 2:** Available reporting guidelines for diagnostic test accuracy research

	Primary diagnostic test accuracy studies	Systematic reviews of diagnostic test accuracy studies
Full-text articles	STARD 2015 [7, 8]	PRISMA-DTA [11, 12]
Journal and conference abstracts	STARD for Abstracts [26]	PRISMA-DTA for Abstracts [11, 13]
Prospective registration	STARD for Registration [33]	Not yet available

Prospective registration of biomedical studies is increasingly encouraged to reduce unnecessary duplicate research efforts, increase transparency, and prevent selective reporting [30]. Where registration of clinical trials of interventions has become commonplace and a requirement for many institutions and journals, researchers evaluating medical tests less often register their study protocol [31, 32]. To improve this, STARD for Registration was developed, providing guidance for informative registration of primary test accuracy studies in trial registries such as clinicaltrials.gov [33]. Systematic reviews should also be prospectively registered before data extraction starts, which can be done in PROSPERO [34], or, alternatively, full protocols and other research materials can be uploaded on online platforms such as Open Science Framework (available at https://osf.io/).

For systematic reviewers, QUADAS-2 (Quality Assessment of Diagnostic Accuracy Studies) provides a tool for the assessment of potential sources of bias or applicability concerns within four domains of primary test accuracy studies [35]. These domains were previously identified as the main sources of quality concerns in test accuracy studies and cover (1) patient selection, (2) the index test under evaluation, (3) the reference standard used, and (4) the flow of patients and the timing of testing. The Cochrane Handbook for Diagnostic Test Accuracy Reviews provides specific guidance for each step in the review process such as developing criteria for including studies, searching for studies, and assessing methodological quality (by applying QUADAS-2) [36]. Later in the potential process of adopting medical tests in clinical practice, developers of clinical guidelines of diagnostic tests and strategies may need to grade the quality of evidence and strength of recommendations, for example by using GRADE [37].

## Discussion: *Diagnostic and Prognostic Research* and reporting guidelines

Incomplete reporting is a significant and avoidable source of research waste [1, 2]. To improve this situation for test accuracy research, reporting guidelines such as STARD 2015, STARD for Abstracts, PRISMA-DTA, and PRISMA-DTA for Abstracts are available. These guidelines are particularly relevant for *Diagnostic and Prognostic Research*, because the journal aims at publishing high-quality diagnostic research addressing studies of medical tests and markers, including systematic reviews thereof. *Diagnostic and Prognostic Research* advocates complete and transparent reporting of research and explicitly highlights in the submission guidelines that “using these guidelines to write the report, completing the checklist, and constructing a flow diagram are likely to optimize the quality of reporting and make the peer review process more efficient.” Therefore, authors are required to upload a populated reporting checklist from the applicable reporting guidelines during the submission process, and editors are instructed to ensure that this is done. There is evidence that such editorial policies improve adherence to reporting guidelines [38], and hence, we encourage journals to consider implementing them if not already in place.

## Data Availability

Not applicable

## References

[1] Glasziou P, Altman DG, Bossuyt P, Boutron I, Clarke M, Julious S, Michie S, Moher D, Wager E (2014). Reducing waste from incomplete or unusable reports of biomedical research. Lancet.

[2] Moher D, Glasziou P, Chalmers I, Nasser M, Bossuyt PM, Korevaar DA, Graham ID, Ravaud P, Boutron I (2016). Increasing value and reducing waste in biomedical research: who’s listening?. Lancet.

[3] Korevaar DA, van Enst WA, Spijker R, Bossuyt PM, Hooft L (2014). Reporting quality of diagnostic accuracy studies: a systematic review and meta-analysis of investigations on adherence to STARD. Evid Based Med.

[4] Altman DG, Simera I (2016). A history of the evolution of guidelines for reporting medical research: the long road to the EQUATOR Network. Journal of the Royal Society of Medicine.

[5] Schulz KF, Altman DG, Moher D (2010). CONSORT 2010 statement: updated guidelines for reporting parallel group randomised trials. BMJ.

[6] Collins GS, Reitsma JB, Altman DG, Moons KG (2015). Transparent reporting of a multivariable prediction model for individual prognosis or diagnosis (TRIPOD): the TRIPOD statement. BMJ.

[7] Bossuyt PM, Reitsma JB, Bruns DE, Gatsonis CA, Glasziou PP, Irwig L, Lijmer JG, Moher D, Rennie D, de Vet HC (2015). STARD 2015: an updated list of essential items for reporting diagnostic accuracy studies. BMJ.

[8] Cohen JF, Korevaar DA, Altman DG, Bruns DE, Gatsonis CA, Hooft L, Irwig L, Levine D, Reitsma JB, de Vet HC (2016). STARD 2015 guidelines for reporting diagnostic accuracy studies: explanation and elaboration. BMJ open.

[9] Korevaar DA, Cohen JF, Reitsma JB, Bruns DE, Gatsonis CA, Glasziou PP, Irwig L, Moher D, de Vet HCW, Altman DG, Hooft L, Bossuyt PMM (2016). Updating standards for reporting diagnostic accuracy: the development of STARD 2015. Res Integr Peer Rev.

[10] Moher D, Liberati A, Tetzlaff J, Altman DG (2009). Preferred reporting items for systematic reviews and meta-analyses: the PRISMA statement. PLoS Medicine.

[11] McInnes MDF, Moher D, Thombs BD, McGrath TA, Bossuyt PM, Clifford T, Cohen JF, Deeks JJ, Gatsonis C, the P-DTAG (2018). Preferred reporting items for a systematic review and meta-analysis of diagnostic test accuracy studies: the PRISMA-DTA statement. JAMA.

[12] Salameh JP, Bossuyt PM, McGrath TA, Thombs BD, Hyde CJ, Macaskill P, Deeks JJ, Leeflang M, Korevaar DA, Whiting P (2020). Preferred reporting items for systematic review and meta-analysis of diagnostic test accuracy studies (PRISMA-DTA): explanation, elaboration, and checklist. BMJ.

[13] Cohen JF, Deeks JJ, Hooft L, Salameh JP, Korevaar DA, Gatsonis C, Hopewell S, Hunt HA, Hyde CJ, Leeflang MM, et al. Preferred reporting items for journal and conference abstracts of systematic reviews and meta-analyses of diagnostic test accuracy studies (PRISMA-DTA for Abstracts): checklist, explanation, and elaboration. BMJ. 2021;372:n265. 10.1136/bmj.n265. 10.1136/bmj.n265 PMC7957862 33722791

[14] Hopewell S, Clarke M, Moher D, Wager E, Middleton P, Altman DG, Schulz KF (2008). CONSORT for reporting randomized controlled trials in journal and conference abstracts: explanation and elaboration. PLoS Med.

[15] Cohen JF, Korevaar DA, Boutron I, Gatsonis CA, Hopewell S, McInnes MDF, Moher D, von Elm E, Bossuyt PM (2020). Reporting guidelines for journal and conference abstracts. J Clin Epidemiol.

[16] Heus P, Reitsma JB, Collins GS, Damen J, Scholten R, Altman DG, Moons KGM, Hooft L (2020). Transparent reporting of multivariable prediction models in journal and conference abstracts: TRIPOD for Abstracts. Ann Int Med.

[17] Scherer RW, Langenberg P, von EE (2007). Full publication of results initially presented in abstracts. Cochrane Datab Syst Rev.

[18] Korevaar DA, Cohen JF, de Ronde MW, Virgili G, Dickersin K, Bossuyt PM (2015). Reporting weaknesses in conference abstracts of diagnostic accuracy studies in ophthalmology. JAMA Ophthalmol.

[19] Korevaar DA, Cohen JF, Hooft L, Bossuyt PM (2015). Literature survey of high-impact journals revealed reporting weaknesses in abstracts of diagnostic accuracy studies. J Clin Epidemiol.

[20] Berwanger O, Ribeiro RA, Finkelsztejn A, Watanabe M, Suzumura EA, Duncan BB, Devereaux PJ, Cook D (2009). The quality of reporting of trial abstracts is suboptimal: survey of major general medical journals. J Clin Epidemiol.

[21] Ochodo EA, de Haan MC, Reitsma JB, Hooft L, Bossuyt PM, Leeflang MM (2013). Overinterpretation and misreporting of diagnostic accuracy studies: evidence of “spin”. Radiology.

[22] McGrath TA, McInnes MDF, van Es N, Leeflang MMG, Korevaar DA, Bossuyt PMM (2017). Overinterpretation of research findings: evidence of “spin” in systematic reviews of diagnostic accuracy studies. Clin Chem.

[23] Boutron I, Dutton S, Ravaud P, Altman DG (2010). Reporting and interpretation of randomized controlled trials with statistically nonsignificant results for primary outcomes. JAMA.

[24] Whiting PF, Rutjes AW, Westwood ME, Mallett S (2013). A systematic review classifies sources of bias and variation in diagnostic test accuracy studies. J Clin Epidemiol.

[25] Korevaar DA, Wang J, van Enst WA, Leeflang MM, Hooft L, Smidt N, Bossuyt PM (2015). Reporting diagnostic accuracy studies: some improvements after 10 years of STARD. Radiology.

[26] Cohen JF, Korevaar DA, Gatsonis CA, Glasziou PP, Hooft L, Moher D, Reitsma JB, de Vet HC, Bossuyt PM (2017). Group S: STARD for Abstracts: essential items for reporting diagnostic accuracy studies in journal or conference abstracts. BMJ.

[27] Beller EM, Glasziou PP, Altman DG, Hopewell S, Bastian H, Chalmers I, Gotzsche PC, Lasserson T, Tovey D (2013). PRISMA for Abstracts: reporting systematic reviews in journal and conference abstracts. PLoS Med.

[28] McGrath TA, Alabousi M, Skidmore B, Korevaar DA, Bossuyt PMM, Moher D, Thombs B, McInnes MDF (2017). Recommendations for reporting of systematic reviews and meta-analyses of diagnostic test accuracy: a systematic review. Syst Rev.

[29] Salameh JP, McInnes MDF, Moher D, Thombs BD, McGrath TA, Frank R, Dehmoobad Sharifabadi A, Kraaijpoel N, Levis B, Bossuyt PM (2019). Completeness of reporting of systematic reviews of diagnostic test accuracy based on the PRISMA-DTA reporting guideline. Clin Chem.

[30] Zarin DA, Keselman A (2007). Registering a clinical trial in ClinicalTrials.gov. Chest.

[31] Korevaar DA, Bossuyt PM, Hooft L (2014). Infrequent and incomplete registration of test accuracy studies: analysis of recent study reports. BMJ Open.

[32] Altman DG (2014). The time has come to register diagnostic and prognostic research. Clinical chemistry.

[33] Korevaar DA, Hooft L, Askie LM, Barbour V, Faure H, Gatsonis CA, Hunter KE, Kressel HY, Lippman H, McInnes MDF (2017). Facilitating prospective registration of diagnostic accuracy studies: a STARD initiative. Clin Chem.

[34] Booth A, Clarke M, Dooley G, Ghersi D, Moher D, Petticrew M, Stewart L (2012). The nuts and bolts of PROSPERO: an international prospective register of systematic reviews. Syst Rev.

[35] Whiting PF, Rutjes AW, Westwood ME, Mallett S, Deeks JJ, Reitsma JB, Leeflang MM, Sterne JA, Bossuyt PM (2011). QUADAS-2: a revised tool for the quality assessment of diagnostic accuracy studies. Ann Int Med.

[36] Deeks JJ, Wisniewski S, Davenport C, Deeks JJ, Bossuyt PM, Gatsonis CA, The Cochrane Collaboration (2013). Chapter 4: guide to the contents of a Cochrane Diagnostic Test Accuracy Protocol. Cochrane handbook for systematic reviews of diagnostic test accuracy. Version 1.0.0.

[37] Schunemann HJ, Oxman AD, Brozek J, Glasziou P, Jaeschke R, Vist GE, Williams JW, Kunz R, Craig J, Montori VM (2008). Grading quality of evidence and strength of recommendations for diagnostic tests and strategies. BMJ.

[38] Turner L, Shamseer L, Altman DG, Weeks L, Peters J, Kober T, Dias S, Schulz KF, Plint AC, Moher D (2012). Consolidated standards of reporting trials (CONSORT) and the completeness of reporting of randomised controlled trials (RCTs) published in medical journals. Cochrane Datab Syst Rev.

